# Supplementary material for: Intermittent Fasting versus Continuous Calorie Restriction: Which Is Better for Weight Loss?
Source: Nutrients. 2022 Apr 24;14(9):1781. doi: 10.3390/nu14091781 (PMC9099935; doi:10.3390/nu14091781)
Supplement: Supplementary file 1 [file nutrients-14-01781-s001.zip › nutrients-1681248-supplementary.pdf]

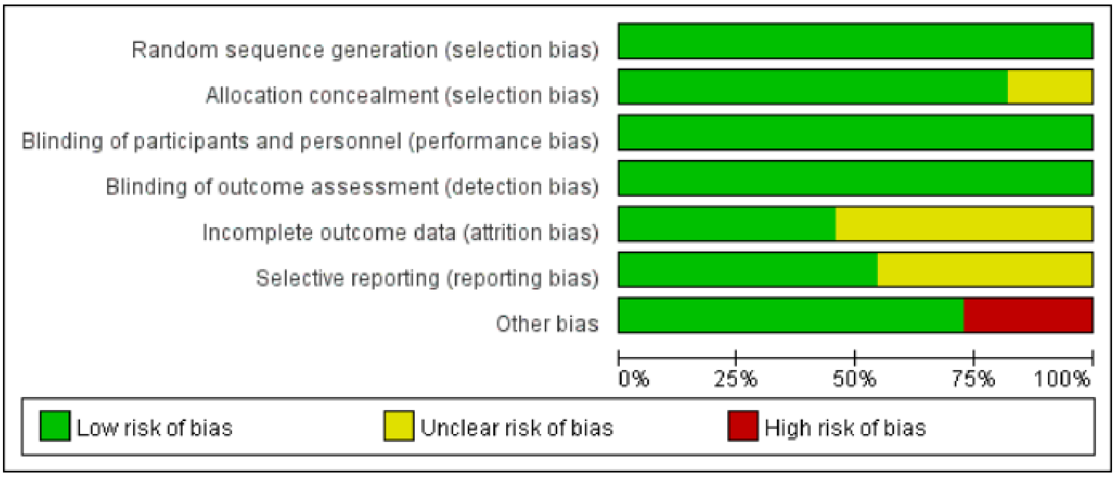

Figure S1. Risk of bias graph: review authors' judgements about each risk of bias item presented as percentages across all included studies.

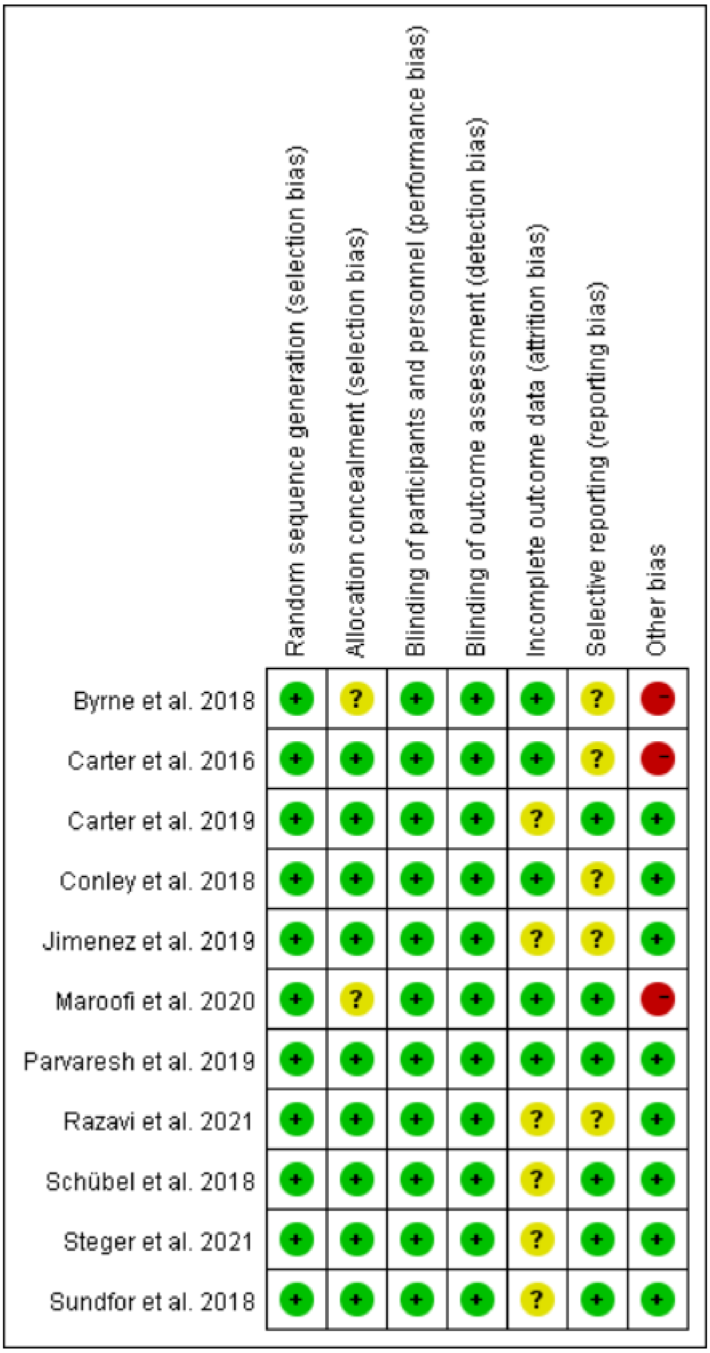

Figure S2.Risk of bias summary: review authors' judgements about each risk of bias item for each included study.
